# Ambient Temperature and Stroke Occurrence: A Systematic Review and Meta-Analysis

**DOI:** 10.3390/ijerph13070698

**Published:** 2016-07-12

**Authors:** Xia Wang, Yongjun Cao, Daqing Hong, Danni Zheng, Sarah Richtering, Else Charlotte Sandset, Tzen Hugh Leong, Hisatomi Arima, Shariful Islam, Abdul Salam, Craig Anderson, Thompson Robinson, Maree L. Hackett

**Affiliations:** 1 The George Institute for Global Health and Royal Prince Alfred Hospital, the University of Sydney, P.O. Box M201, Missenden Road, Sydney, NSW 2050, Australia; xwang@georgeinstitute.org.au (X.W.); dhong@georgeinstitute.org.au (D.H.); dzheng@georgeinstitute.org.au (D.Z.); srichtering@georgeinstitute.org.au (S.R.); hugh-96@hotmail.com (T.H.L.); sIslam@georgeinstitute.org.au (S.I); asalam@georgeinstitute.org.au (A.S.); mhackett@georgeinstitute.org.au (M.L.H.); 2 Sydney Medical School, the University of Sydney, Sydney, NSW 2006, Australia; 3 Department of Neurology, the Second Affiliated Hospital of Soochow University, No. 1055, Sanxiang Rd., Suzhou 215004, China; 4 Division of Nephrology, Sichuan Academy of Medical Sciences & Sichuan Provincial People’s Hospital, Chengdu 610072, China; 5 Department of Neurology, Oslo University Hospital, Oslo 0424, Norway; else@sandset.net; 6 Department of Preventive Medicine and Public Health, Faculty of Medicine, Fukuoka University, Fukuoka 814-0180, Japan; harima@georgeinstitute.org.au; 7 Department of Cardiovascular Sciences and NIHR Biomedical Research Unit for Cardiovascular Diseases, University of Leicester, Leicester LE1 7RH, UK; tgr2@leicester.ac.uk; 8 College of Health and Wellbeing, the University of Central Lancashire, Preston PR1 2HE, UK

**Keywords:** stroke, weather, temperature, systematic review

## Abstract

Biologically plausible associations exist between climatic conditions and stroke risk, but study results are inconsistent. We aimed to summarize current evidence on ambient temperature and overall stroke occurrence, and by age, sex, and variation of temperature. We performed a systematic literature search across MEDLINE, Embase, PsycINFO, CINAHL, Web of Science, and GEOBASE, from inception to 16 October 2015 to identify all population-based observational studies. Where possible, data were pooled for meta-analysis with Odds ratios (OR) and corresponding 95% confidence intervals (CI) by means of the random effects meta-analysis. We included 21 studies with a total of 476,511 patients. The data were varied as indicated by significant heterogeneity across studies for both ischemic stroke (IS) and intracerebral hemorrhage (ICH). Pooled OR (95% CI) in every 1 degree Celsius increase in ambient temperature was significant for ICH 0.97 (0.94–1.00), but not for IS 1.00 (0.99–1.01) and subarachnoid hemorrhage (SAH) 1.00 (0.98–1.01). Meta-analysis was not possible for the pre-specified subgroup analyses by age, sex, and variation of temperature. Change in temperature over the previous 24 h appeared to be more important than absolute temperature in relation to the risk of stroke, especially in relation to the risk of ICH. Older age appeared to increase vulnerability to low temperature for both IS and ICH. To conclude, this review shows that lower mean ambient temperature is significantly associated with the risk of ICH, but not with IS and SAH. Larger temperature changes were associated with higher stroke rates in the elderly.

## 1. Introduction

Estimates from the Global Burden of Diseases, Injuries, and Risk Factors Study (GBD 2010) ranked stroke as the second most common cause of death [1] and the third most common cause of disability-adjusted life-years (DALYs) [2] worldwide in 2010. In 2010, the absolute number of people with first stroke (16.9 million), stroke survivors (33 million), stroke-related deaths (5.9 million), and DALYs lost (102 million) were extremely high, having increased significantly since 1990 (increases of 68%, 84%, 26% and 12%, respectively). With the shift of demographics around the world towards an increasingly ageing population, the global stroke burden is predicted to continue to rise in the coming years [3], and therefore, every effort must be taken to minimize the impact of stroke.

An influence of climate upon cerebrovascular risk is biologically plausible. Mild Earth surface cooling has been shown to increase platelet count, blood viscosity, arterial pressure and plasma cholesterol [4]. Also, since lower temperatures induce peripheral vasoconstriction, arterial blood pressure rises in colder months and decreases in hotter months within the same individual [5,6]. High blood pressure is an important risk factor of all stroke subtypes: ischemic stroke (IS) [7], intracerebral hemorrhage (ICH) [8,9], and subarachnoid hemorrhage (SAH) [10].

Studies of the influence of meteorological factors upon stroke occurrence have yielded inconsistent results. Some authors have suggested that low temperature and/or a large change in temperature/air pressure are associated with stroke incidence [11,12,13,14,15,16,17,18,19], whereas others have found no such relationship [20,21,22]. This may be due to research limitations such having a study population from a small geographic region, little variation in weather parameters [13,15,16,19,23], recruitment from a single hospital [11,16,17,18], small sample size [12,14], relatively short study period over a single year [12,16], or undifferentiated stroke subtypes [13]. There is also influence of diverse meteorological parameters and different statistical analysis techniques. One review [24] synthesized evidence of short-term mean ambient temperature on stroke occurrence, mortality and morbidity, however, it included hospital-based studies which may introduce selection bias [21,25].

Therefore, we conducted a systematic review of population-based stroke studies to determine whether ambient temperature is associated with hospital admission for stroke, and whether any association was influenced by age, sex, and variation of temperature.

## 2. Materials and Methods

The protocol for this study was registered with the international prospective register of systematic reviews (PROSPERO)—CRD42015026364. The systematic review was reported following Meta-analysis of Observational Studies in Epidemiology (MOOSE) guidelines [26]. There were no language restrictions.

### 2.1. Study Eligibility Criteria

Population or community-based, and hospital registry studies that explicitly stated consecutive patient recruitment from within clearly defined geographical boundaries and of a minimum one-year duration (to ensure all seasons were represented) were included. Patients aged 18 years and over, of any race or gender with a clinical or imaging (computed tomography [CT] or magnetic resonance imaging [MRI]) diagnosis of first-ever or recurrent stroke, regardless of pathological subtype, were included. Hospital-based studies and studies with broad vascular outcomes, no specific results for stroke, with less than 50% stroke patients, or with fewer than 100 patients were excluded. In addition, studies that cited on weather data from unofficial sources (e.g., any temperature website) were excluded.

### 2.2. Databases and Sources

A comprehensive search strategy (Supplementary Material: Search Strategy), developed in consultation with a university librarian, neurologists, and epidemiologists, was used to address the unique features and indexing of each of the five electronic databases (MEDLINE, Embase, PsycINFO, CINAHL, and Web of Science), that were searched from inception to 16th October 2015. GEOBASE was also searched to capture any relevant studies that might have been published in the geographical/ meteorological rather than medical literature.

In order to capture important “grey literature”, the websites of the following organizations were reviewed for relevant reports: World Health Organization; European Union; Health Effects Institute (USA); Environmental Protection Agency (USA); National Institutes of Health (USA); Department of Health (UK); Department for Environment, Food, and Rural Affairs (UK); Department of Health (Australia); Department of the Environment (Australia); Ministry of Health of the People’s Republic of China; Ministry of Environmental Protection of the People’s Republic of China. As well as searching for original studies, the reference lists of any relevant reviews appearing in their reports were examined. For any data presented in reviews but not reported in the paper, authors were contacted to request original data.

### 2.3. Data Collection and Extraction

X.W. scrutinized the titles and abstracts, and excluded clearly irrelevant references; this process was double-checked by S.R. X.W. and D.H. extracted data independently from the included studies using EpiData; any disagreements were resolved by a third author (D.Z.).

### 2.4. Data Analysis

Six items were used to define quality criteria to appraise included studies [27]: (A) presence of clear hypotheses; (B) prospective study design; (C) description of the population, at least including its size, and the gender ratio; (D) stroke assessed by CT, MRI or angiography, cerebrospinal fluid examination or autopsy; (E) a clear description of the meteorological determinants investigated, when possible including the unit of measurement; and (F) description of other risk factors for stroke. Articles were defined as ‘high quality’ when at least five of these criteria were satisfied.

We pooled the results of studies in which an effect estimate was presented as a regression coefficient, percentage change (PC), relative risk (RR) or odds ratio (OR). We converted the regression coefficient and PC to RR using the equations: RR = $e^{\beta }$ and RR = 1 + PC, respectively. Thereafter, all RRs were converted to ORs using the equation: OR = RR/[(1 − P0) + (P0 × RR)], where P0 = the incidence of non-exposed group. Because population stroke incidence fulfilled a poisson distribution, so it was a small probability event, we assumed OR = RR. The degree of heterogeneity was calculated using the *I*^2^-index. ORs from the individual studies were pooled with the random effects method because of the large degree of variation in the overall effect estimates between studies. Otherwise, a narrative review of studies was presented. Exploring the association between ambient temperature and stroke occurrence is complex, which may be due to that fact that stroke is a heterogeneous condition [28], so stroke subtypes were reported separately.

## 3. Results

**Figure 1:**
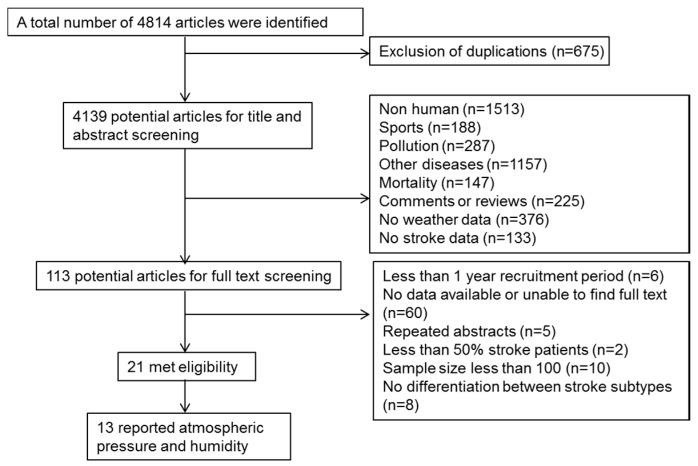
Flow chart of literature search.

Of 4814 references obtained after execution of the search strategy, 113 remained after screening titles and abstracts for relevance (Figure 1). Twenty-one studies (476,511 patients) that satisfied the eligibility criteria were included in the review (Table 1). Of the 21 studies, two were community-based, 14 were population-based, and five were based on stroke registries. Seven studies were defined as high quality as they met five of the quality criteria, another seven met four and the remainer met less than four of the quality criteria. In brief, studies were conducted in 12 countries, across five continents; only two studies were conducted in the Southern Hemisphere. Seven studies were from countries with latitude less than 30 degrees and 14 were between 30 and 60 degrees. 

### 3.1. Ischemic Stroke

#### 3.1.1. Mean, Minimum and Maximum Temperature

**Figure 2:**
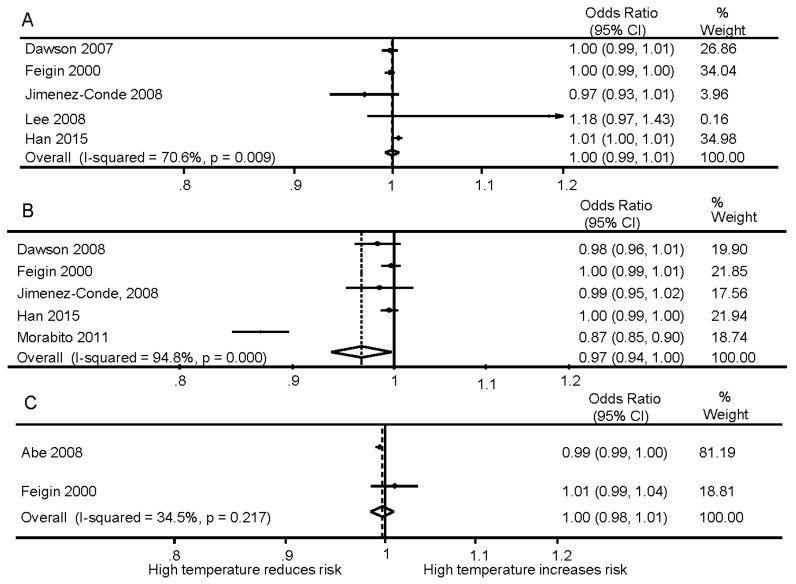
Meta-analysis of ambient mean temperature and stroke: (**A**) ischemic stroke; (**B**) intracerebral haemorrhage; (**C**) subarchnoid haemorrhage.

Eight studies, which are summarized in Supplementary Tables S1 and S2, involving a total of 290,154 patients, reported data on mean ambient temperature. Pooled estimate (5 studies) showed no significant association between ambient temperature and IS (OR 1.00, 95% CI 0.99–1.01) (Figure 2).

**Figure 3:**
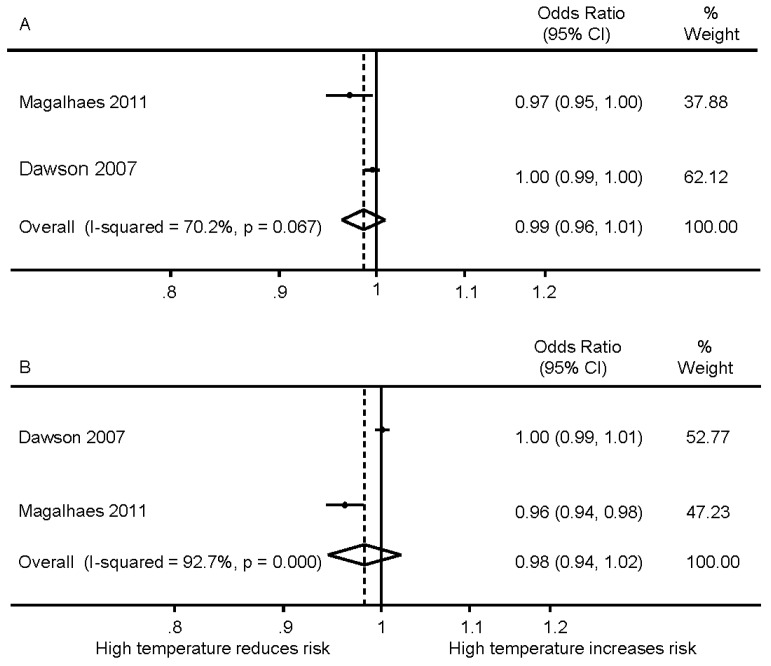
Meta-analysis of ambient temperature and ischemic stroke: (**A**) daily minimum temperature; (**B**) daily maximum temperature.

Two studies reported minimum and maximum temperature, and pooled results again showed no significant association between ambient minimum and maximum temperature and IS; OR 0.99 (0.96–1.01) and 0.98 (0.94–1.02), respectively (Figure 3).

#### 3.1.2. Subgroup by Sex and Age

Four studies reported subgroup analysis by sex; two studies finding a statistically significant association between low mean ambient temperature and IS risk in women, one reporting a significant association between warmer mean temperature and IS risk in men, and one study reporting a significant association between low mean temperature and IS risk in both women and men (Supplementary Table S3). Three studies reported subgroup analysis by age, and all found a stronger association between low ambient mean temperature and IS in older patients (Supplementary Table S4).

#### 3.1.3. Temperature Change

Seven studies investigated the association between temperature change in the previous 24 h and stroke occurrence. Two high quality studies reported associations between larger changes in mean ambient temperature and an increase in IS hospital admissions, but not for daily mean temperature per se (Table S5). Data from one clinical stroke registry in Glasgow (United Kingdom) with 5723 IS patients from 1990 to 2005, found that every 1 degree Celsius increase in mean temperature during the preceding 24 h was associated with a 2.1% increase in IS admissions (*p* = 0.004) [30]. Similar results were seen in a population-based study including 45,787 IS patients in Tuscany (Italy) from 1997 to 2007, where every degree Celsius increase in temperature difference over 24 h was associated with a 1.5% increase in IS admissions (*p* < 0.0001) [33].

### 3.2. Intracerebral Hemorrhage

#### 3.2.1. Mean, Minimum and Maximum Temperature

Eleven studies assessed associations between ICH and temperature; six studies reporting that ICH occurred more frequently on colder days (Supplementary Tables S6 and S7). One study conducted in Siberia (Russia) found that the ICH occurrence was associated with mild ambient temperature (classified as −1.9 °C to 7.2 °C), but not low temperature (≤2 °C). In the pooled estimate of four studies, increased mean ambient temperature was significantly associated with ICH risk; OR 0.97, (0.94–1.00 (Figure 2)).

#### 3.2.2. Subgroup by Sex and Age

Three studies reported inconsistent results by sex, probably reflecting the small sample sizes of 51 to 799 individuals (Supplementary Table S8). Similarly to IS, all studies reported a statistically significant association between low ambient temperatures and ICH in the elderly (Supplementary Table S9).

#### 3.2.3. Temperature Change

A statistically significant increase in ICH risk with larger temperature change, whether in the previous 24 h or monthly fluctuation, was reported in four out of eight studies (Supplementary Table S10).

### 3.3. Subarachnoid Hemorrhage

Results for an association between mean ambient temperature and SAH were inconsistent (Supplementary Table S11). Data could only be pooled from two of five studies with a combined OR 1.00 (0.98–1.01, Figure 2). Two studies reported the association between the temperature change over the previous 24 h and SAH occurrence; one reporting a larger temperature change being associated with SAH risk (Supplementary Table S12).

## 4. Discussion

In this comprehensive review, a consistent association of daily mean, minimum or maximum ambient temperature with IS, and SAH occurrence was not identified. In particular, conclusions were limited by the inability to pool all studies because of a lack of crude data and heterogeneity in data presentation. However, we found that lower mean ambient temperature was significantly associated with ICH risk. Subgroup analysis showed that lower temperature increased IS risk in women, and older age appeared to increase vulnerability to temperature for both IS and ICH. Furthermore, change in ambient temperature over the previous 24 h appeared to be more important, especially in relation to ICH risk.

Sex and age differences are apparent in stroke incidence, severity and prognosis [44,45]. With regards to temperature, the effects of sex on the association between ambient temperature and IS risk are in line with previous reports [25,38,46], but there is absence of evidence of gender differences for ICH and SAH. A recent meta-analysis reported that the association between ambient temperature and stroke occurrence was irrelevant for those who were 18 to 64 years old, but it is stronger among the elderly (≥65 years). They also observed opposite associations for males and females [24].

There are plausible physiological explanations for the impact of ambient temperature on stroke occurrence. It is postulated that vasoconstriction occurs in response to lower ambient temperature in an attempt to divert blood flow to central organs. This in turn would increase systemic vascular resistance and cause an increase in blood pressure [5,47]. Also, mean systolic and diastolic blood pressure tend to be higher during colder months, and exposure to cold can exacerbate hypertension in predisposed individuals [48]. This may reflect increasing systemic vascular resistance and oxygen demand, and also less effective adaptation to cold due to autonomic nervous system dysregulation in hypertensive individuals [49]. Finally, vasoconstriction may lead to an excess of blood flow in vital vascular beds, such as the cerebral vasculature, which theoretically could increase the risk of haemorrhage [28].

There are several limitations in this review. First, although we assessed the quality of the included articles, we were unable to incorporate quality scores in our meta-analysis because of the overall poor reporting of results. Secondly, many included studies had small sample sizes, and results were not adjusted for confounders. Factors such as air conditioning and heating systems, together with the direct effect of the weather on behaviour, such as alcohol intake and activity level are crucial confounding factors that are virtually impossible to adjust for in retrospective analyses. Thirdly, we were not able to pool results for the majority of studies, due to a lack of crude data and/ or inconsistent presentation of meteorological data. For example, Feigin et al. reported a non-significant result for temperature as a continuous variable, but when analyzed as a categorical variable, low temperature (≤−2 °C) was significantly associated with a lower IS risk compared to high temperature (≥7.3 °C); RR 1.32 (1.05–1.66). It is also noticeable that the pooled data showed high heterogeneity. Fourthly, we were not able to assess publication bias since a limited number of studies were included. Finally, direct comparison between studies was difficult because of different study design and differences in weather across regions; for example, does a 1 °C difference in a warm climate mean the same in a cold climate? These heterogeneous findings reinforce the need for locality-specific data to aid the understanding of the temperature effects on stroke occurrence, and to assess for differential effects in stroke subtype.

## 5. Conclusions

In conclusion, available data on the evidence for an association between ambient temperature and incidence of stroke are conflicting. This review shows that lower mean ambient temperature is significantly associated with ICH risk, but not with IS and SAH. Importantly, further research is needed to clarify the extent and nature of any relationship between temperature and stroke in different geographical areas, and in respect of stroke subtype.

## Figures and Tables

**Table 1:** Characteristics of studies included in the review.

Paper ID	Author and Year of Publication	Title	Country & City/Region	Latitude	Year(s) of Study	Sample Size	Age (Mean, Year) Female (%)	Only First-Ever Stroke	Stroke Subtype	Study Type	Study Quality
1	Abe 2008 [29]	Effects of meteorological factors on the onset of subarachnoid hemorrhage: a time-series analysis	Japan, Tokyo	35.6833° N	2005	1729	63.3 Female (60%)	No	SAH	Population study	ACDEF
2	Dawson 2008 [30]	Associations between meteorological variables and acute stroke hospital admissions in the west of Scotland	United Kingdom, Glasgow	55.8580° N	1990–2005	6389	71.2 Female (53%)	No	IS and ICH	Stroke registry	ACDE
3	Feigin 2000 [25]	A population-based study of the associations of stroke occurrence with weather parameters in Siberia, Russia (1982–1992)	Russia, Siberia	61.0137° N	1982–1992	2208	Age range: 25–74 Female (57%)	Yes	IS, ICH and SAH	Stroke registry	ABCEF
4	Jimenez-Conde 2008 [17]	Weather as a trigger of stroke: daily meteorological factors and incidence of stroke subtypes	Spain, Barcelona	41.3833° N	2001–2003	1286	Not reported	No	IS and ICH	Population	ABDE
5	Lee 2008 [31]	Seasonal variation in ischemic stroke incidence and association with climate, a six-year population-based study	China, Taiwan	25.0330° N	1998–2003	168,977	Age range: 20–84	No	IS	Population	AE
6	Magalhaes 2011 [32]	Are stroke occurrence and outcome related to weather parameters? Results from a population-based study in Northern Portugal	Portugal, Porto	41.1621° N	1998–2000	462	All ages Female (62%)	Yes	IS and ICH	Stroke registry	ACDEF
7	Morabito 2011 [33]	Innovative approaches helpful to enhance knowledge on weather-related stroke events over a wide geographical area and a large population	Italy, Tuscany	43.3500° N	1997–2007	112,870	All ages	No	IS, ICH and SAH	Hospital registry	ACDE
8	Han 2015 [34]	Effect of seasonal and monthly variation in weather and air pollution factors on stroke incidence in Seoul, Korea	South Korea, Seoul	37.5667° N	2004–2013	3001	Age >19 Female (49%)	No	IS and ICH	Stroke registry	ACDEF
9	Chen 1995 [16]	Weather and stroke in a subtropical area: Ilan, Taiwan	Taiwan, Ilan	24.7570° N	1991	517	All ages Female (39%)	No	IS, ICH and SAH	Population	ACDE
10	Fang 2012 [19]	Ambient temperature and spontaneous intracerebral haemorrhage: a crossectional analysis in Tainan, Taiwan	China, Taiwan	22.9999° N	08/2006–07/2008	933	62 Female (39%)	No	ICH	Stroke registry	ACDEF
11	Gomes 2014 [35]	Triggering of stroke by ambient temperature variation: a case-crossover study in Maputo, Mozambique	Maputo, Mozambique	25.9500° S	08/2005–07/2006	593	58.8 Female (48%)	Yes	IS and ICH	Population	ABDE
12	Lai 2014 [36]	The association between meteorological parameters and aneurysmal subarachnoid hemorrhage: a nationwide analysis	USA, 41 states	38.8833° N	2001–2010	16,970	Median: 53 (IQR 34–72)	No	SAH	Population	ADE
13	Lejeune 1994 [37]	Association of occurrence of aneurysmal bleeding with meteorological variations in the north of france	France, North France region	47.0000° N	1989–1991	283	49.1 Female (53%)	No	SAH	Community	ABE
14	Matsumoto 2010 [38]	Cumulative effects of weather on stroke incidence: a multi-community cohort study in Japan	Japan, 12 communities	35.6833° N	04/1992–07/2002	450	Age ≥30 in 1 community, 40–69 in 11 communities	Yes	IS, ICH and SAH	Population	ABDEF
15	Nakaguchi 2008 [39]	Prediction of the incidence of spontaneous intracerebral hemorrhage from meteorological data	Japan Shin-ichi	34.3319° N	01/2001–12/2003	164	All ages	No	ICH	Community	ADE
16	Shinkawa 1990 [40]	Seasonal variation in stroke incidence in Hisayama, Japan	Japan, Hisayama	33.6468° N	11/1961–10/1985	308	Age ≥40, 74 Female (49%)	Yes	IS, ICH and SAH	Population	ABCDEF
17	Sobel 1987 [41]	Stroke in the Lehigh Valley: seasonal variation in incidence rates	United States, Lehigh Valley	40.6646° N	07/1982–12/1983	1944	All ages Female (51%)	No	IS, ICH and SAH	Hospital registry	ABCE
18	Tsementzis 1991 [42]	Seasonal variation of cerebrovascular diseases	United Kingdom, West Midlands Region	52.489471° N	1973–1980	12,262	All ages Female (53%)	No	IS, ICH and SAH	Hospital registry	AE
19	Wang 2009 [15]	Temperature variation and emergency hospital admissions for stroke in Brisbane, Australia, 1996-2005	Australia, Brisbane	27.4667° S	1996–2005	12387	All ages	No	IS and ICH	Population	ACE
20	Oyoshi 1999 [43]	Relationship between aneurysmal subarachnoid hemorrhage and climatic conditions in the subtropical region, Amami-Oshima, in Japan	Japan, Amami-Oshima	28.2500° N	1986–1996	210	All ages, 64.3	No	SAH	Hospital registry	AE
21	Goggins 2012 [23]	Weather, season, and daily stroke admissions in Hong Kong	China, Hong Kong	22.2783° N	1999-–2006	130,962	≥35	No	IS, ICH and SAH	Hospital registry	ACDE

Abbreviations: IS: ischemic stroke; ICH: intracerebral haemorrhage; SAH: subarachnoid haemorrhage; N: north; S: south.

## Supplementary Materials

The following are available online at www.mdpi.com/1660-4601/13/7/698/s1, Table S1: Meta-analysis for ambient temperature and ischemic stroke (IS), Table S2: Studies included in systematic review of mean temperature and ischemic stroke (IS), Table S3: Studies included in systematic review of mean temperature and ischemic stroke (IS) by sex, Table S4: Studies included in systematic review of ambient temperature and ischemic stroke (IS) by age, Table S5: Studies included in systematic review of temperature change and ischemic stroke (IS), Table S6: Meta analysis for mean temperature and intracerebral hemorrhage (ICH), Table S7: Studies only included in systematic review of mean temperature and intracerebral hemorrhage (ICH), Table S8: Studies included in systematic review of mean temperature and intracerebral hemorrhage (ICH) by sex, Table S9: Studies included in systematic review of ambient temperature and intracerebral hemorrhage (ICH) by age, Table S10: Studies included in systematic review of temperature change and intracerebral hemorrhage (ICH), Table S11: studies for mean temperature and subarachnoid hemorrhage (SAH), Table S12: Studies included in systematic review of ambient temperature and subarachnoid hemorrhage (SAH).
