# Supplementary material for: Ambient Temperature and Stroke Occurrence: A Systematic Review and Meta-Analysis
Source: Int J Environ Res Public Health. 2016 Jul 12;13(7):698. doi: 10.3390/ijerph13070698 (PMC4962239; doi:10.3390/ijerph13070698)
Supplement: Supplementary file 1 [file ijerph-13-00698-s001.pdf]

Supplementary Materials: Ambient Temperature and Stroke Occurrence: A Systematic Review and Meta-Analysis

Xia Wang, Yongjun Cao, Daqing Hong, Danni Zheng, Sarah Richtering, Else Charlotte Sandset, Tzen Hugh Leong, Hisatomi Arima, Shariful Islam, Abdul Salam, Craig Anderson Thompson Robinson and Maree L. Hackett

Supplementary Material: Search Strategy

MEDLINE

Stroke

cerebrovascular disorders/ or exp basal ganglia cerebrovascular disease/ or exp brain ischemia/ or exp carotid artery diseases/ or exp intracranial arterial diseases/ or exp intracranial arteriovenous malformations/ or exp "intracranial embolism and thrombosis"/ or exp intracranial hemorrhages/ or stroke/ or exp brain infarction/ or vasospasm, intracranial/ or vertebral artery dissection/

(stroke$ or poststroke or post-stroke or cerebrovasc$ or brain vasc$ or cerebral vasc$ or cva$ or apoplex$ or SAH).tw.

((brain$ or cerebr$ or cerebell$ or intracran$ or intracerebral) adj3 (isch?emi$ or infarct$ or thrombo$ or emboli$ or occlus$)).tw.

((brain$ or cerebr$ or cerebell$ or intracerebral or intracranial or subarachnoid) adj3 (h?emorrhag$ or h?ematoma$ or bleed$)).tw.

exp paresis/ or exp Gait Disorders, Neurologic/

(hemipleg$ or hemipar$ or pares?s or paretic).tw.

1 or 2 or 3 or 4 or 5 or 6

Weather

exp weather/ or exp climate/ or exp meteorological concepts/

weather or climate

(climat$ or season$ or humid$ or latitud$).tw.

(temperature$ adj3 (hot$ or cold$ or ambient or extreme$ or atmospher$ or variation$ or chang$ or absolute or range$ or fall$ or rise$ or fluctuat$)).tw

((atmospher$ or air or barometric) adj3 pressur$).tw

(snow$ or sun$ or rain$ or freez$ or precipitation$ or meteorolog$).tw.

8 or 9 or 10 or 11 or 12 or 13

Study type

Epidemiologic studies/or Exp case control studies/ or Exp cohort studies/ or Case control.tw. or (cohort adj (study or studies)).tw. or Cohort analy$.tw. or (Follow up adj (study or studies)).tw. or (observational adj (study or studies)).tw. or Longitudinal.tw. or Retrospective.tw. or Cross sectional.tw. or Cross-sectional studies/

Human/

7 and14 and 15 and 16

EMBASE

Stroke

'cerebrovascular disease' or 'basal ganglion hemorrhage'/exp or 'brain hematoma'/exp or 'brain hemorrhage'/exp or 'brain infarction'/exp or 'brain ischemia'/exp or 'carotid artery disease'/exp or 'cerebral artery disease' or 'cerebrovascular accident'/exp or 'intracranial aneurysm'/exp or 'occlusive cerebrovascular disease'/exp or 'stroke unit' or 'stroke patient'

(stroke$ or poststroke or post-stroke or cerebrovasc$ or brain vasc$ or cerebral vasc$ or cva$ or apoplex$ or SAH):ab or (stroke$ or poststroke or post-stroke or cerebrovasc$ or brain vasc$ or cerebral vasc$ or cva$ or apoplex$ or SAH):ti

((brain$ or cerebr$ or cerebell$ or intracran$ or intracerebral) near/3 (isch?emi$ or infarct$ or thrombo$ or emboli$ or occlus$)):ti or ((brain$ or cerebr$ or cerebell$ or intracran$ or intracerebral) near/3 (isch?emi$ or infarct$ or thrombo$ or emboli$ or occlus$)):ab

((brain$ or cerebr$ or cerebell$ or intracerebral or intracranial or subarachnoid) near/3 (h?emorrhag$ or h?ematoma$ or bleed$)):ti or ((brain$ or cerebr$ or cerebell$ or intracerebral or intracranial or subarachnoid) near/5 (h?emorrhag$ or h?ematoma$ or bleed$)):ab

'paresis'/exp or 'neurologic gait disorder'/exp

(hemipleg$ or hemipar$ or paresis or pareses or paretic):ab or (hemipleg$ or hemipar$ or paresis or pareses or paretic):ti

1 or 2 or 3 or 4 or 5 or 6

Weather

'weather'/exp or 'climate'/exp or 'season'/exp or 'air temperature'/exp or 'environmental temperature'/exp or 'high temperature'/exp or 'low temperature'/exp or 'atmospheric pressure'/exp or 'humidity'/exp or weather or climate or meteorological phenomena

(climat$ or season$ or humid$ or latitud$):ab or (climat$ or season$ or humid$ or latitud$):ti

(temperature$ near/3 (hot$ or cold$ or ambient or extreme$ or atmospher$ or variation$ or chang$ or absolute or range$ or fall$ or rise$ or fluctuat$)):ab or (temperature$ near/3 (hot$ or cold$ or ambient or extreme$ or atmospher$ or variation$ or chang$ or absolute or range$ or fall$ or rise$ or fluctuat$)):ti

((atmospher$ or air or barometric) near/3 pressure):ab or ((atmospher$ or air or barometric) near/3 pressure):ti

(snow$ or sun$ or rain$ or freez$ or precipitation$ or meteorolog$):ab or (snow$ or sun$ or rain$ or freez$ or precipitation$ or meteorolog$):ti

8 or 9 or 10 or 11 or 12

Study type

'clinical study' or 'case control study' or 'family study' or 'longitudinal study' or 'retrospective study'

'prospective study' not 'randomized controlled trials'

'cohort'

Cohort near (analysis or study or studies)

(Case control near (study or studies)):ab or (Case control near (study or studies)):ti

(follow up near (study or studies)):ab or (follow up near (study or studies)):ti

(observational near (study or studies)):ab or (observational near (study or studies)):ti

(epidemiologic$ near (study or studies)):ab or (epidemiologic$ near (study or studies)):ti

(cross sectional near (study or studies)):ab or (cross sectional near (study or studies)):ti

14 or 15 or 16 or or 17 or 18 or 19 0r 20 or 21 or 22

CINAHL

Stroke

Cerebrovascular Disorders OR (MH "Basal Ganglia Cerebrovascular Disease+") OR (MH "Carotid Artery Diseases+") OR (MH "Cerebral Ischemia+") OR (MH "Arteriovenous Malformations+") OR (MH "Cerebral Vasospasm") OR (MH "Intracranial Arterial Diseases+") OR (MH "Intracranial Embolism and Thrombosis+") OR (MH "Intracranial Hemorrhage+") OR (MH "Stroke+") OR (MH "Vertebral Artery Dissections") or (MH "Stroke Patients") OR (MH "Stroke Units")

TI (stroke* or poststroke or post-stroke or cerebrovasc* or brain vasc* or cerebral vasc* or cva* or apople* or SAH) or AB (stroke* or poststroke or post-stroke or cerebrovasc* or brain vasc* or cerebral vasc* or cva* or apople* or SAH)

(TI (brain or cerebr* or cerebell* or intracran* or intracerebral) or AB (brain or cerebr* or cerebell* or intracran* or intracerebral)) and (TI (ischemi* or ischaemi* or infarct* or thrombo* or emboli* or occlus*) or AB (ischemi* or ischaemi* or infarct* or thrombo* or emboli* or occlus*))

(TI (brain* or cerebr* or cerebell* or intracerebral or intracranial or subarachnoid) or AB (brain* or cerebr* or cerebell* or intracerebral or intracranial or subarachnoid)) and (TI (haemorrhage* or hemorrhage* or haematoma* or hematoma* or bleed*) or AB (haemorrhage* or hemorrhage* or haematoma* or hematoma* or bleed*))

TI (hemipleg* or hemipar* or paresis or pareses or paretic) or AB (hemipleg* or hemipar* or paresis or pareses or paretic)

1 or 2 or 3 or 4 or 5 or 6

Weather

(MH "Meteorological Factors+") or (MH "atmosphere+") OR (MH "Atmospheric Pressure+") or (MH "Weather+") OR (MH "Temperature+"))

(MM "Cold") OR (MM "Heat") OR (MM "Humidity") OR (MM "Rain") OR (MM "Snow") or (MM "Seasons")

AB (climat$ or season$ or humid$ or latitud$) or TI (climat$ or season$ or humid$ or latitud$)

AB (temperature$ N3 (hot$ or cold$ or ambient or extreme$ or atmospher$ or variation$ or chang$ or absolute or range$ or fall$ or rise$ or fluctuat$)) or TI (temperature$ N3 (hot$ or cold$ or ambient or extreme$ or atmospher$ or variation$ or chang$ or absolute or range$ or fall$ or rise$ or fluctuat$))

TI ((atmospher$ or air or barometric) N3 pressure) or AB ((atmospher$ or air or barometric) N3 pressure)

TI (snow$ or sun$ or rain$ or freez$ or precipitation or meteorolog$) or AB (snow$ or sun$ or rain$ or freez$ or precipitation$ or meteorolog$)
7 OR 8 OR 9 OR 10 OR 11 OR 12

Study type

Prospective studies OR (MH "Case Control Studies+") or Correlational studies or Nonconcurrent prospective studies or Cross sectional studies or TI (cohort N (study or studies)) OR AB (cohort N (study or studies)) OR TI (observational N (study or studies)) OR AB (observational N (study or studies))

Web of Science

Stroke

TS=(stroke OR cva OR cerebrovascular OR ″cerebral vascular“ OR hemipleg* OR paresis OR pareses OR hemipares* OR parapares*)

TS=(paretic OR hemiparetic OR paraparetic OR dystoni*)

TS=((cerebral OR cerebellar OR brain* OR vertebrobasilar) NEAR/3 (infarct* OR isch$emi* OR thrombo* OR emboli* OR apoplexy))

TS=((cerebral OR brain* OR subarachnoid) NEAR/3 (haemorrhage OR haemorrhage OR haematoma OR hematoma OR bleed*))

#4 OR #3 OR #2 OR #1

Weather

TS=(weather or climat* or season* or humid* or latitud* or snow* or rain* or precipitation*)

TS=(temperature* NEAR/3 (hot* or cold* or ambient or extreme* or atmospher* or variation* or chang* or absolute or range* or fall$ or rise$ or fluctuat$))

TS=((atmospher* or air or barometric) NEAR/3 pressur*)

#6 OR #7 OR #8

#5 and #9

PsycINFO (Ovid)

Stroke

cerebrovascular disorders/ or cerebral hemorrhage/ or exp cerebral ischemia/ or cerebral small vessel disease/ or cerebrovascular accidents/ or subarachnoid hemorrhage/

(stroke or poststroke or post-stroke or cerebrovasc$ or brain vasc$ or cerebral vasc$ or cva$ or apoplex$ or SAH).tw.

((brain$ or cerebr$ or cerebell$ or intracran$ or intracerebral) adj3 (isch?emi$ or infarct$ or thrombo$ or emboli$ or occlus$)).tw.

((brain$ or cerebr$ or cerebell$ or intracerebral or intracranial or subarachnoid) adj3 (h?emorrhage$ or h?ematoma$ or bleed$)).tw.

hemiparesis/ or hemiplegia/

(hemipleg$ or hemipar$ or paresis or paretic).tw.

or/1-6

Weather

exp seasonal variations/ exp Atmospheric Conditions/

(weather or climat$ or season$ or humid$ or latitud$).tw.

(temperature$ adj3 (hot$ or cold$ or ambient or extreme$ or atmospher$ or variation$ or chang$ or absolute or range$ or fall$ or rise$ or fluctuat$)).tw

((atmospher$ or air or barometric) adj3 pressur$).tw

(snow$ or sun$ or rain$ or freez$ or precipitation$ or meteorolog$).tw.

8 or 9 or 10 or 11 or 12 or 13

7 and 14

Geobase

Stroke

(({nervous system disorder} WN CV OR {nervous system disorder} WN RGI) OR ({stroke} WN CV OR {stroke} WN RGI))

((stroke$ or poststroke or post-stroke or cerebrovasc$ or brain vasc$ or cerebral vasc$ or cva$ or apoplex$ or SAH) WN KY)

$brain near/3 $ischemi or $brain near/3 $ischaemi or $brain near/3 $infarct or $brain near/3 $thrombo or $brain near/3 $emboli or $brain near/3 $occlus

$cerebr near/3 $ischemi or $cerebr near/3 $ischaemi or $cerebr near/3 $infarct or $cerebr near/3 $thrombo or $cerebr near/3 $emboli or $cerebr near/3 $occlus

$cerebell near/3 $ischemi or $cerebell near/3 $ischaemi or $cerebell near/3 $infarct or $cerebell near/3 $thrombo or $cerebell near/3 $emboli or $cerebell near/3 $occlus

$intracran near/3 $ischemi or $intracran near/3 $ischaemi or $intracran near/3 $infarct or $intracran near/3 $thrombo or $intracran near/3 $emboli or $intracran near/3 $occlus

intracerebral near/3 $ischemi or intracerebral near/3 $ischaemi or intracerebral near/3 $infarct or intracerebral near/3 $thrombo or intracerebral near/3 $emboli or intracerebral near/3 $occlus

$brain near/3 $hemorrhage or $cerebr near/3 $hemorrhage or $cerebell near/3 $hemorrhage or $intracran near/3 $hemorrhage or intracerebral near/3 $hemorrhage

$brain near/3 $ hematoma or $cerebr near/3 $ hematoma or $cerebell near/3 $ hematoma or $intracran near/3 $ hematoma or intracerebral near/3 $hematoma

$brain near/3 $bleed or $cerebr near/3 $bleed or $cerebell near/3 $bleed or $intracran near/3 $bleed or intracerebral near/3 $bleed

((hemipleg$ or hemipar$ or pares?s or paretic or Neurologic Gait Disorders) WN KY)

Weather

(({atmospheric and meteorological phenomena} WN CV OR {atmospheric and meteorological phenomena} WN RGI) OR ({climate} WN CV OR {climate} WN RGI) OR ({weather} WN CV OR {weather} WN RGI) OR ({severe weather} WN CV OR {severe weather} WN RGI))

((weather or climat$ or season$ or humid$ or latitud$) WN KY)

(({air temperature} WN CV OR {air temperature} WN RGI))

(({atmospheric pressure} WN CV OR {atmospheric pressure} WN RGI))

(({season} WN CV OR {season} WN RGI) OR ({seasonality} WN CV OR {seasonality} WN RGI))

((snow or sun or rain or freeze or precipitation) WN KY)

Table S1. Meta-analysis for ambient temperature and ischemic stroke (IS).

Temperature Variable Studied	Paper ID	Authors and Year of Publication	Number of IS Cases Reported	Statistical ANALYSIS method	Variables Controlled for in the Model	Overall Conclusion	Comments
Daily mean temperature	2	Dawson 2007	5723	Negative binomial model	Study year, season and day of the week	No statistically significant association	
	3	Feigin 2000	1929	Poisson regression	Age, solar and geomagnetic activity, season	Statistically significant association between colder ambient temperature and first IS occurrence	
	4	Jimenez-Conde 2008	732	Logistic regression	Atmospheric pressure and humidity	No statistically significant association	Non-lacunar stroke

Monthly mean temperature	5	Lee 2008	168977	Auto-regressive integrated moving average analysis	Seasonality, month and trend	No statistically significant association
	8	Han 2015	2202	Poisson generalized linear regression	Temperature range, humidity, PM_{10} and NO_2	Statistically significant association between warmer temperature and IS risk
Daily minimum temperature	2	Dawson 2007	5723	Negative binomial model	Study year, season and day of the week	No statistically significant association
	6	Magalhaes 2011	348	Poisson regression	Not reported	Statistically significant association between colder temperature and IS risk
Daily maximum temperature	2	Dawson 2007	5723	Negative binomial model	Study year, season and day of the week	No statistically significant association
	6	Magalhaes 2011	348	Poisson regression	Not reported	Statistically significant association between colder temperature and IS risk

Table S2. Studies included in systematic review of mean temperature and ischemic stroke (IS).

Paper ID	Authors and Year of Publication	Number of IS Cases Reported	Temperature Variable Studied	Statistical Analysis Method	Variables Controlled for in the Model	Overall Conclusion
21	Goggins 2012	107505	Daily mean temperature (lag 0–13 days)	Poisson generalized additive models	Humidity, solar radiation, rainfall, air pressure, pollutants, flu consultation rates, day of week, holidays, time trend and seasonality	Statistically significant association between colder temperature and IS risk for below 22 °C temperature
17	Sobel 1987	1306	Monthly mean temperature	Pearson correlation	N/A	Statistically significant association between colder temperature and IS risk
18	Tsementzis 1991	434	Monthly mean temperature	Linear regression	Humidity, pressure, rain, and sunshine	Statistically non-significant association between colder temperature and IS risk
3	Feigin 2000	1929	Daily mean temperature: low (≤−2.0 °C), mild (−1.9 to 7.2 °C) vs. high (≥7.3.°C, reference variable)	Poisson regression	Season, geomagnetic, sun activity, and age	Statistically significant association between colder temperature and first IS risk

Table S3. Studies included in systematic review of mean temperature and ischemic stroke (IS) by sex.

Paper ID	Authors and Year of Publication	Number of IS Cases Reported	Temperature Variable Studied	Statistical Analysis Method	Variables Controlled for in the Model	Overall Conclusion
14	Matsumoto 2010	290	Daily mean temperature	Multilevel logistic regression	Age, obesity, current smoking status, total cholesterol, systolic blood pressure, diabetes, and other meteorological parameters (including rainfall and sunlight)	Among women, statistically significant association between colder temperature and IS risk
16	Shinkawa 1990	223	Monthly mean temperature	Stepwise multivariate regression	Not reported	Among women, statistically significant association between colder temperature and IS risk
8	Han 2015	2202	Monthly mean temperature	Poisson generalized linear regression	Temperature range, humidity, PM_{10} and NO_2	Among men, statistically significant association between warmer temperature and IS risk
21	Goggins 2012	107505	Daily mean temperature (lag 0-13 days)	Poisson generalized additive models	Humidity, solar radiation, rainfall, air pressure, pollutants, flu consultation rates, day of week, holidays, time trend and seasonality	Statistically significant association between colder temperature and IS risk below 22°C for both sex, however the association was stronger for female sex

Table S4. Studies included in systematic review of ambient temperature and ischemic stroke (IS) by age.

Paper ID	Authors and Year of Publication	Number of IS Cases Reported	Temperature Variable Studied	Statistical Analysis Method	Variables Controlled for in the Model	Overall Conclusion
19	Wang 2009	6743	Daily maximum and minimum temperature	Generalized estimating equations (GEE) model	Humidity and air pollutants including PM_{10}, NO_2, O_3 and SO_2	For aged≥65 in winter, statistically significant association between colder maximum temperature and IS admission
8	Han 2015	2202	Monthly mean temperature	Poisson generalized linear regression	Temperature range, humidity, PM_{10} and NO_2	For aged≥60, statistically significant association colder temperature and IS risk
21	Goggins 2012	107505	Daily mean temperature (lag 0-13 days)	Poisson generalized additive models	Humidity, solar radiation, rainfall, air pressure, pollutants, flu consultation rates, day of week, holidays, time trend and seasonality	As age increases, the association between colder temperature and IS risk becomes stronger, there was a particularly large jump in relative risk for those aged >85

Table S5. Studies included in systematic review of temperature change and ischemic stroke (IS).

Paper ID	Authors and Year of Publication	Number of IS Cases Reported	Temperature Variable Studied	Statistical Analysis Method	Variables Controlled in the Model	Overall Conclusion
2	Dawson 2007	5723	Daily change in mean temperature over preceding 24 h and 48 h	Negative binomial model	Study year, season and day of the week	Every 1 °C increase in mean temperature during the preceding 24 h was significantly associated with a 2.1% increase in IS admissions
3	Feigin 2000	1929	Any change towards increasing or decreasing mean temperature vs. no change,	Poisson regression	None (univariate model)	No statistically significant association
11	Gomes 2014	351	Daily maximum decrease in minimum temperature ≥2.4 °C vs. <2.4 °C	Conditional logistic regression	Humidity and precipitation	No statistically significant association

6	Magalhaes 2011	348	Diurnal temperature range in previous 24 h, 7 and 14 days	Poisson regression	Not reported	No statistically significant association
7	Morabito 2011	45787	Temperature difference in previous 24 h	Generalized linear model	Years, seasons, days of the week, celebrations, summer decrement of population	Statistically significant association between larger change and IS risk
8	Han 2015	2202	Diurnal temperature range (monthly)	Poisson generalized linear regression	Temperature range, humidity, PM_{10} and NO_2	No statistically significant association
4	Jimenez-Conde 2008	1043	Variations of mean, minimum, and maximum temperature in previous 24 h	Logistic regression	Atmospheric pressure, humidity	No statistically significant association

Table S6. Meta analysis for mean temperature and intracerebral hemorrhage (ICH).

Paper ID	Authors and Year of Publication	Number of ICH Cases Reported	Statistical Analysis Method	Variables Controlled in the Model	Overall Conclusion
2	Dawson 2007	666	Poisson regression	Study year, season and day of the week	No statistically significant association
3	Feigin 2000	215	Poisson regression	None	No statistically significant association
4	Jimenez-Conde 2008	243	Logistic regression	Atmospheric pressure, humidity	No statistically significant association
8	Han 2015	799	Poisson generalized linear regression	Temperature range, humidity, PM_{10} and NO_2	No statistically significant association
7	Morabito 2011	12478	Poisson generalized linear model	Years, seasons, days of the week, celebrations, summer decrement of population	Statistically significant association between colder temperature and ICH risk

Table S7. Studies only included in systematic review of mean temperature and intracerebral hemorrhage (ICH).

Paper ID	Authors and Year of Publication	Number of ICH Cases Reported	Weather Parameter Studied	Analysis Model	Variables Controlled	Overall Conclusion
9	Chen 1995	170	Daily mean temperature (>27.3 °C; 17.3–27.3 °C; <17.3 °C)	Linear regression model	Atmospheric pressure	Statistically significant association between colder temperature and ICH risk
16	Shinkawa 1990	51	Monthly mean temperature	Multiple linear regression	Not reported	Statistically significant association between colder temperature and ICH risk
17	Sobel 1987	130	Monthly mean temperature	Pearson correlation	N/A	Statistically significant association between colder temperature and ICH risk
10	Fang 2012	933	Monthly mean temperature and temperature range	Linear regression model	Not reported	Statistically significant association between colder temperature and ICH risk
3	Feigin 2000	215	Daily mean temperature: low (<−2.0 °C), mild (−1.9 to 7.2 °C) vs. high (>7.3 °C)	Posson regression	Season, geomagnetic, sun activity, and age	Statistically significant association between ICH occurrence and mild ambient temperature
6	Magalhaes 2011	91	Daily minimum and maximum temperature in previous 7 and 14 days	Poisson regression	Not reported	No statistically significant negative association
15	Nakaguchi 2008	164	Daily maximum and minimum temperature	Mann-Whitney's U-test		ICH tends to occur on days with lower maximum air temperature.
19	Wang 2009	1835	Daily maximum and minimum temperature	Generalized estimating equations (GEE) model	Humidity, PM_{10}, NO_2, O_3 and SO_2	Statistically significant association between colder temperature with maximum temperature
4	Jimenez-Conde 2008	243	Mean, maximum and minimum temperature	Logistic regression	Atmospheric pressure and humidity	No statistically significant association

Table S8. Studies included in systematic review of mean temperature and intracerebral hemorrhage (ICH) by sex.

Paper ID	Authors and Year of Publication	Number of ICH Cases Reported	Weather Parameter Studied	Analysis Model	Variables Controlled	Overall Conclusion
14	Matsumoto 2010	102	Daily mean temperature	Multilevel logistic regression	Age, obesity, smoking status, total cholesterol, systolic blood pressure, diabetes, and other meteorological parameters	Statistically significant association between colder temperature and ICH risk among females but not males
16	Shinkawa 1990	51	Monthly mean temperature	Stepwise multivariate analysis	Not reported	Statistically significant association between cloder temperature and ICH risk among males but not females
8	Han 2015	799	Monthly mean temperature	Poisson generalized linear regression	Temperature range, humidity, PM_{10} and NO_2	No statistically significant association

Table S9. Studies included in systematic review of ambient temperature and intracerebral hemorrhage (ICH) by age.

Paper ID	Authors and Year of Publication	Number of ICH Cases Reported	Weather Parameter Studied	Analysis Model	Variables Controlled	Overall Conclusion
19	Wang 2009	1835	Daily maximum and minimum temperature	Generalized estimating equations (GEE) model	Humidity and air pollutants	Statistically significant association between ICH risk and colder maximum temperatures in patients aged ≥65 and for patients <65 in summer
7	Morabito 2011	12478	Daily mean temperature	Poisson generalized linear model	Years, seasons, days of the week, celebrations, summer decrement of population	Statistically significant association between colder temperature and ICH risk among patients ≥65
8	Han 2015	799	Monthly mean temperature	Poisson generalized linear regression	Temperature range, humidity, PM_{10} and NO_2	Statistically significant association between colder temperature and ICH risk among aged ≥60

Table S10. Studies included in systematic review of temperature change and intracerebral hemorrhage (ICH).

Paper ID	Authors and Year of Publication	Number of ICH Cases Reported	Weather Parameter Studied	Analysis Model	Variables Controlled	Overall Conclusion
10	Fang 2012	933	Monthly temperature fluctuation	Linear regression model	None	Statistically significant association between larger change and ICH risk
3	Feigin 2000	215	Daily change in mean temperature towards increasing or decreasing vs. no change	Poisson regression	Season, geomagnetic, sun activity, and age	No statistically significant association
11	Gomes 2014	242	Daily maximum decrease in minimum temperature ≥2.4 °C vs. <2.4 °C	Conditional logistic regression	Humidity and precipitation	Statistically significant association between larger change and ICH risk
6	Magalhaes 2011	91	Diurnal temperature range in previous 24 h, 7 and 14 days	Poisson regression	N/A	Statistically significant association with larger temperature range in the previous 24 h
16	Shinkawa 1990	51	Intradiurnal temperature	Stepwise multivariate analysis	Not reported	Statistically significant association between larger change and ICH risk
8	Han 2015	799	Diurnal temperature range	Poisson generalized linear regression	Temperature range, humidity, PM_{10} and NO_2	No statistically significant association
7	Morabito 2011	12478	Temperature change in the previous 24 h	Poisson generalized linear model	Years, seasons, days of the week, celebrations, summer decrement of population	No statistically significant association
4	Jimenez-Conde 2008	243	Mean, maximum and minimum temperature variation in the previous 24 h	Logistic regression	Atmospheric pressure and humidity	No statistically significant association

Table S11. studies for mean temperature and subarachnoid hemorrhage (SAH).

Paper ID	Authors and Year of Publication	Number of SAH Cases Reported	Temperature Variable Studied	Statistical Analysis Method	Variables Controlled in the Model	Overall Conclusion
1	Abe 2008	1729	Hourly mean temperature	Autoregressive integrated moving average model	Atmospheric pressure, humidity	Statistically significant association between colder temperature and SAH risk
3	Feigin 2000	64	Daily mean temperature	Poisson regression	Season, geomagnetic, sun activity, and age	No statistically association
12	Lai 2014	16970	Daily mean temperature	Linear regression	Sex	Statistically significant association between warmer temperature and SAH risk
20	Oyoshi 1999	210	Monthly mean, maximum, minimum temperature	Pearson correlation	N/A	No statistically significant association
18	Tsementzis 1991	430	Monthly mean temperature	Linear regression	Humidity, pressure, rain, and sunshine	No statistically significant association

Table S12. Studies included in systematic review of ambient temperature and subarachnoid hemorrhage (SAH).

Paper ID	Authors and Year of Publication	Number of SAH Cases Reported	Temperature Variable Studied	Statistical Analysis Method	Variables Controlled in the Model	Overall Conclusion
3	Feigin 2000	64	Low (<−2.0), mild (−1.9 to 7.2) vs. high (>7.3) mean temperature. Any change towards increasing or decreasing vs. no change,	Poisson regression	Season, geomagnetic, sun activity, and age	No statistically significant association
13	Lejeune, 1994	283	Reduction of 1 of maximal temperature on the day of SAH; reduction of 1 of maximal temperature on the day before SAH	Logistic regression	Atmospheric pressure, humidity and sunshine duration	Statistically significant association between larger temperature change and SAH risk

© 2016 by the authors; licensee MDPI, Basel, Switzerland. This article is an open access article distributed under the terms and conditions of the Creative Commons by Attribution (CC-BY) license (http://creativecommons.org/licenses/by/4.0/).
